# Combined effects of electrical muscle stimulation and cycling exercise on cognitive performance

**DOI:** 10.3389/fphys.2024.1408963

**Published:** 2024-05-17

**Authors:** Soichi Ando, Yuka Ishioka, Sari Kambayashi, Kosuke Kano, Mami Fujibayashi, Joseph T. Costello, Mizuki Sudo

**Affiliations:** ^1^ Graduate School of Informatics and Engineering, The University of Electro-Communications, Chofu, Tokyo, Japan; ^2^ Faculty of Informatics and Engineering, The University of Electro-Communications, Chofu, Tokyo, Japan; ^3^ Faculty of Agriculture, Setsunan University, Hirakata, Japan; ^4^ School of Sport, Health and Exercise Science, University of Portsmouth, Portsmouth, United Kingdom; ^5^ Physical Fitness Research Institute, Meiji Yasuda Life Foundation of Health and Welfare, Shinjuku, Tokyo, Japan

**Keywords:** cognition, voluntary exercise, combined stressors, combined exercise, reaction time

## Abstract

The purpose of this study was to investigate whether a combination of electrical muscle stimulation (EMS) and cycling exercise is beneficial for improving cognitive performance. Eighteen participants (7 females and 11 males) performed a Go/No-Go task before and 2 min after i) cycling exercise (EX), ii) a combination of EMS and cycling (EMS + EX) and iii) a control (rest) intervention in a randomized controlled crossover design. In the EX intervention, the participants cycled an ergometer for 20 min with their heart rate maintained at ∼120 beats·min^-1^. In the EMS + EX intervention, the participants cycled an ergometer simultaneously with EMS for 20 min, with heart rate maintained at ∼120 beats·min^-1^. In the Control intervention, the participants remained at rest while seated on the ergometer. Cognitive performance was assessed by reaction time (RT) and accuracy. There was a significant interaction between intervention and time (*p* = 0.007). RT was reduced in the EX intervention (*p* = 0.054, matched rank biserial correlation coefficient = 0.520). In the EMS + EX intervention, RT was not altered (*p* = 0.243, Cohen’s d = 0.285) despite no differences in heart rate between the EX and EMS + EX interventions (*p* = 0.551). RT was increased in the Control intervention (*p* = 0.038, Cohen’s d = −0.529). These results indicate that combining EMS and cycling does not alter cognitive performance despite elevated heart rate, equivalent to a moderate intensity. The present findings suggest that brain activity during EMS with cycling exercise may be insufficient to improve cognitive performance when compared to exercise alone.

## 1 Introduction

A growing body of evidence suggests that acute physical exercise at low-to moderate-intensity has the potential to improve cognitive performance (Lambourne and Tomporowski, 2010; Chang et al., 2012; Ando et al., 2020; McMorris, 2021). Nevertheless, it remains to be elucidated how a single bout of exercise leads to cognitive improvements. To understanding how acute exercise improves cognitive performance, it is critical to identify what triggers cognitive improvement. Voluntary exercise induces a myriad of physiological changes that originate centrally (e.g., brain activity associated with central motor command), peripherally (e.g., muscle contraction and resultant metabolic/hormonal changes), and under cardiovascular command (Williamson et al., 2006; Fisher et al., 2015). On the contrary, electrical muscle stimulation (EMS), which is also known as neuromuscular electrical stimulation, involuntarily induces muscle contraction. EMS does not require central motor or cardiovascular command. Thus, EMS allows to isolate the physiological changes derived from peripheral muscle contraction during exercise.

Emerging evidence has examined the benefits of EMS on cognitive performance. For example, two studies indicated that EMS does not improve cognitive performance (Miyamoto et al., 2018; Ando et al., 2024), while another recent study indicated that EMS improved cognitive performance in the Stroop congruent task and short-term memory performance, but not in the Stroop incongruent task or the trail making test (Descollonges et al., 2024). Hence, the findings concerning the effects of acute EMS on cognitive performance are conflicting, but physiological changes induced by EMS alone may be insufficient to improve performance in all cognitive domains. Intriguingly, a follow-up experiment revealed that a combination of EMS and arm cranking exercise improves cognitive performance (Ando et al., 2024). These results may emphasize that brain activity associated with voluntary exercise is necessary for cognitive improvements induced by acute exercise.

Previously, other group showed that a combination of EMS to lower limb muscles and cycling exercise has additional effects on metabolic and cardiovascular responses (Watanabe et al., 2014; Watanabe et al., 2021). In our previous study, EMS was applied to inactive muscle groups (i.e., lower limb muscles) during arm cranking (Ando et al., 2024), and it remains to be elucidated whether a combination of EMS applied to active muscle groups and cycling exercise improves cognitive performance. The combination of EMS and cycling exercise is practically applicable to those who have difficulties with exercising at moderate to higher intensities. A combination of EMS and cycling allows to increase exercise intensity with less amount of voluntary exercise. Given that exercise at moderate intensity is more likely to improve cognitive performance (Lambourne and Tomporowski, 2010; Chang et al., 2012; Ando et al., 2020; McMorris, 2021), empirical evidence on combined effects of EMS and cycling exercise on cognitive performance is required to provide novel insights into promoting cognitive and brain health.

In the present study, based on the assumption that brain activity associated with voluntary exercise is important for cognitive improvement in response to acute exercise, we hypothesized that a combination of EMS and cycling exercise improves cognitive performance. Accordingly, using a randomized controlled crossover design, we tested the hypothesis that a combination of EMS and cycling exercise would improve cognitive performance.

## 2 Materials and methods

### 2.1 Participants

Eighteen participants (7 females and 11 males, age: 21.5 ± 1.2 years, height: 169.6 ± 10.2 cm, weight: 64.3 ± 10.8 kg) were recruited in this study. Sample size was estimated *a priori* based on reaction time (RT), the primary outcome measure, from our unpublished data. Accordingly, a minimum of 15 participants were required to achieve a power of 80% with an alpha of 0.05 based on a Cohen’s d (effect size of 0.4). A total of 18 participant were recruited for the current study to account for potential drop-out. The participants were free from any known cardiovascular, cerebrovascular, or respiratory disease. They were asked to refrain from moderate to vigorous physical activity for 24 h and consuming any food or drink, except water, for 3 h before each experiment. This study was approved by the University of Electro-Communications’ Human Ethics Committee (No: 19,003). The study conformed to the standards set by the latest revision of the Declaration of Helsinki, except for registration in the database. Each participant provided their written informed consent prior to participation.

### 2.2 Experimental procedure

Participants visited the laboratory on four separate occasions. At the initial visit, intensity of EMS was adjusted for each participant. EMS was administered to the abdominal, gluteal, thigh, and leg muscle groups of the participants with an electrical stimulator in a supine position (Auto Tens Pro; Homerion laboratory Co., Ltd, Tokyo, Japan) (Miyamoto et al., 2018; Ando et al., 2021; Ando et al., 2024). The stimulator current waveform was set at a frequency of 4 Hz with a pulse width of 0.25 m. The stimulation intensity was gradually increased and set to the maximum tolerable level as previously described (Hamada et al., 2003; Ando et al., 2021; Ando et al., 2024). The current waveform was designed to exponentially increase the pulse, which reduced pain/discomfort during EMS (Hasegawa et al., 2011).

On the second, third, and fourth visits to the laboratory, the participants performed the experimental interventions with a wash-out of at least 2 days between visits. The order of the interventions was randomized. In all interventions, the participants performed the cognitive task before and 2 min after acute exercise intervention and rest (control). In the Exercise (EX) intervention, the participants cycled an ergometer (75XLIII, Combi Wellness Corporation, Tokyo, Japan) for 20 min while maintaining their heart rate (HR) at ∼120 beats·min^-1^. In the EMS combined with cycling upright exercise (EMS + EX) intervention, the participants cycled the ergometer simultaneously with EMS for 20 min while maintaining their HR at ∼120 beats·min^-1^. Intensity of EMS was adjusted to 80% of the predetermined maximal intensity, which allowed a smooth combination of EMS and cycling exercise. The duration of EMS with voluntary exercise was set at 20 min since an exercise duration of at least 20 min is believed to be required to improve cognitive performance/reaction time (Chang et al., 2012). In the Control intervention, the participants remained seated on the ergometer for 20 min. Ambient temperature was maintained at 22°C–23°C throughout the experiment.

### 2.3 Cognitive task

In the present study, Go/No-Go task was used to evaluate cognitive performance. Details of the cognitive task were described elsewhere (Akagi et al., 2019; Saito et al., 2021; Ando et al., 2023). Briefly, the visual stimuli were controlled using the Presentation software (Presentation ver.19; NeuroBehavioral Systems, Berkeley, CA, USA). Each trial started with a blank screen for 2.5 s, followed by a preparatory stimulus (green square) presentation at the center of the computer screen for 1 s. Then, either Go or No-Go signal (color squares) was randomly presented for 1 s. One block of the Go/No-Go task consisted of 30 Go trials (red and blue) and 30 No-Go trials (yellow and purple). In the Go trial, the participants were asked to press a button of a numeric pad with their right index finger as quickly as possible. In the No-Go trial, they were asked to withhold the response. Cognitive performance was assessed using reaction time (RT, ms) and accuracy (%). Omissions of responses in Go-trials or incorrect responses in No-Go trials were also excluded as an ‘error’. Accuracy was calculated as number of correct responses/total number of trials.

### 2.4 Measurements

HR was monitored with an ear sensor that was connected to the ergometer. At the beginning of the experimental sessions, HR was measured while at rest and seated on a chair. Exercise intensity was gradually increased by 30–32 W min^-1^. Once HR reached at the target value (i.e., 120 beats·min^-1^), exercise intensity was automatically adjusted to maintain the target HR (Komiyama et al., 2015; Komiyama et al., 2016). One of the investigators continuously monitored the HR and manually adjusted the exercise intensity every minute if the HR deviated substantially from the target value. The validity of the ear sensor was confirmed elsewhere (Ando et al., 2015; Komiyama et al., 2015). In the EX and EMS + EX interventions, HR was averaged during exercise intervention after the participants’ HR reached at 120 beats·min^-1^. In the Control intervention, HR was average during the resting period. In the EX and EMS + EX interventions, ratings of perceived exertion (RPE) was measured (Borg 6–20) (Borg, 1982) before exercise interventions and every minute after the HR reached the target level. In the Control intervention, RPE was measured every minute during resting period. Then, RPE during exercise interventions and resting period was averaged.

### 2.5 Statistical analysis

The distribution of data was assessed using descriptive methods (skewness, outliers, and distribution plots) and inferential statistics (Shapiro-Wilk test). We performed a two-way repeated-measures ANOVA [intervention (EX, EMS + EX, and Control) × time (pre vs. post)] on RT, accuracy, HR, and RPE, followed by Bonferroni-corrected paired t-tests for normally distributed data. The Wilcoxon signed rank test was performed for the non-normally distributed data. Effect sizes are presented as eta-squared (η^2^). Cohen’s *d* or matched rank biserial correlation coefficient was also used to report effect sizes. Statistical analyses were performed using the JASP version 18.3 (JASP team, Amsterdam, Netherlands). The degree of freedom was corrected using the Huynh Feldt Epsilon when the assumption of sphericity was violated. Data are expressed as mean ± standard deviation (SD) or median (interquartile range). The significance level was set at *p* < 0.05.

## 3 Results

The peak stimulus intensities of EMS, which were determined on the first day, were 100 ± 30 mA (left thigh), 101 ± 31 mA (right thigh), 61 (40–71) mA (left lower leg), and 55 ± 15 mA (right lower leg). Mean exercise intensity was 67 ± 31 W in the EX intervention and 40 ± 21 W in the EMS + EX intervention, with the intensity greater in the EX intervention (*p* < 0.001).

**FIGURE 1:**
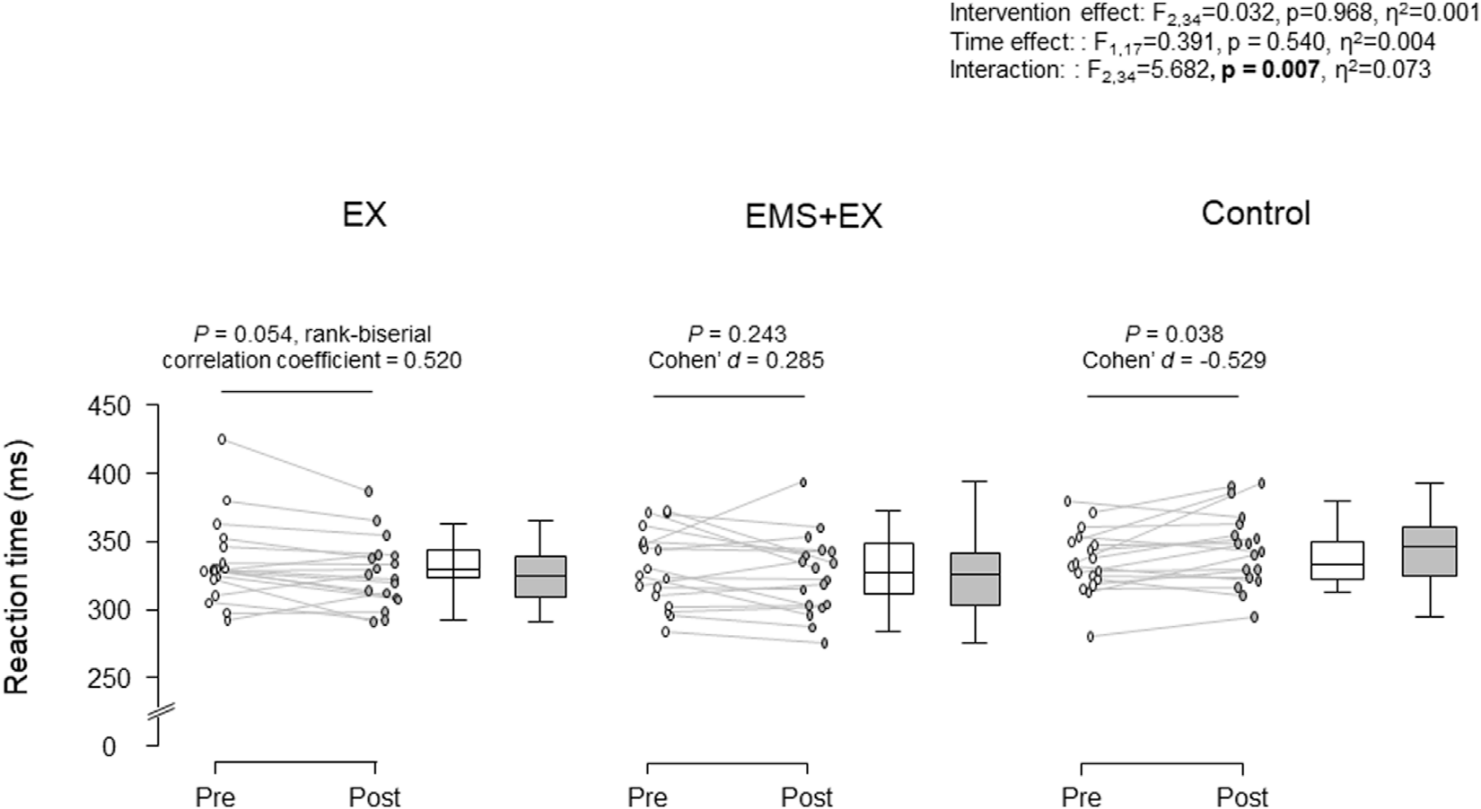
Reaction time in the Exercise (EX), Electrical muscle stimulation combined with cycle exercise (EMS + EX), and Control interventions. Left: individual data, right: the first and third quartiles with the median. Rank-biserial correlation coefficient Cohen’s *d* indicate effect sizes.

Figure 1 shows RT in the EX, EMS + EX, and Control interventions. There was a significant interaction between intervention and time (F_2,34_ = 5.682, *p* = 0.007, η^2^ = 0.073). There were no significant main effects of intervention (F_2,34_ = 0.032, *p* = 0.968, η^2^ = 0.001) or time (F_1,17_ = 0.391, *p* = 0.540, η^2^ = 0.004). Post-hoc analyses revealed that RT was reduced in the EX intervention (*p* = 0.054, matched rank biserial correlation = 0.520, 95% confidence interval (CI) [0.051, 0.802]), but did not change in the EMS + EX intervention (*p* = 0.243, Cohen’s *d* = 0.285, 95% CI [-0.191, 0.752]). RT was increased in the Control intervention (*p* = 0.038, Cohen’s *d* = −0.529, 95% CI [-1.017, −0.028]).

Table 1 shows the accuracy of the cognitive task, HR, and RPE results. No main (intervention: F_2,34_ = 0.406, *p* = 0.670, η^2^ = 0.010, time: F_1,17_ = 4.000, *p* = 0.062, η_p_^2^ = 0.055) or interaction (F_2,34_ = 0.136, *p* = 0.873, η^2^ = 0.002) effects were observed for accuracy. We observed significant main effects of intervention (F_1.270,21.591_ = 192.136, *p* < 0.001, η^2^ = 0.199) and time for HR (F_1,17_ = 889.913, *p* < 0.001, η^2^ = 0.569). We also observed an interaction (F_1.391,23.642_ = 242.119, *p* < 0.001, η^2^ = 0.190). When compared to rest, HR increased during EX, EMS + EX, and the Control interventions (all *p* < 0.001). HR was comparable during EX and EMS + EX interventions (*p* = 0.551). There were significant main effects of intervention (F_2,34_ = 149.400, *p* < 0.001, η^2^ = 0.252) and time of RPE (F_1,17_ = 157.897, *p* < 0.001, η^2^ = 0.411), and an interaction between them (F_2,34_ = 90.127, *p* < 0.001, η^2^ = 0.222). RPE was greater during EX and EMS + EX compared to pre values (all *p* < 0.001).

**TABLE 1:** Accuracy of the cognitive performance, heart rate (HR), and ratings of perceived exertion (RPE) in the Exercise (EX), electrical muscle stimulation (EMS) with exercise (EMS + EX), and Control interventions (n = 18).

Variables	EX		EMS + EX		Control	
	Pre	During/Post	Pre	During/Post	Pre	During/Post
Accuracy	100 (100–100)	100 (98.3–100)	100 (100–100)	100 (98.3–100)	100 (98.8–100)	99.2 (98.3–100)
HR	74.3 ± 7.6	120.4 (120.3–120.8) ***	73.8 ± 6.8	120.5 (120.3–120.6) ***	73.6 ± 6.6	79.6 ± 10.2 ***
RPE	7 (7–7)	11.9 ± 1.7 ***	7 (7–8.5)	12.7 ± 1.7 ***	7(6.3–7)	7 (6.3–7.3)

Values are mean ± standard deviation or median (interquartile range). ***p < 0.001, vs. Pre.

## 4 Discussion

In the present study, we tested the hypothesis that a combination of EMS and cycling exercise improves cognitive performance. Modest improvements in cognitive performance were observed following EX intervention, which is in line with previous studies suggesting that acute exercise is beneficial to cognitive performance (Lambourne and Tomporowski, 2010; Chang et al., 2012; Ando et al., 2020; McMorris, 2021). However, in contrast to our initial hypothesis, cognitive performance did not change in the EMS + EX intervention despite the same HR (∼120 beats·min^-1^). The present findings suggest that a combination of EMS and cycling exercise does not improve cognitive performance, at least under the present experimental interventions.

Increases in HR during voluntary exercise are determined by the balance between the influences of the parasympathetic and sympathetic branches of the autonomic nervous system (White and Raven, 2014). The autonomic responses to exercise are orchestrated by the interactions of several central and peripheral neural mechanisms (Fisher, 2014), which are integrated in the nucleus of the solitary tract in the brain (Raven et al., 2019). Two primary mechanisms activate the sympathetic nervous system during exercise: central command, which is central signal from the higher brain, and exercise pressor reflex, a peripheral reflex arising from the working skeletal muscles (Williamson et al., 2006; Fisher et al., 2015). EMS alone is thought to induce exercise pressor reflex, as shown by modest increases in HR during EMS (Hamada et al., 2004; Ando et al., 2021; Ando et al., 2024). In contrast, during voluntary exercise, both central command and exercise pressor reflex are involved in the autonomic control (Williamson et al., 2006; Fisher et al., 2015). In the present EMS + EX intervention, EMS was added to cycling to increase exercise intensity and reduce the amount of voluntary exercise. Hence, to maintain the same HR level, it can be postulated that the amount of central neural activity associated with voluntary exercise was lower during exercise in the EMS + EX intervention relative to the EX intervention. In the present study, cognitive performance did not improve in the EMS + EX intervention, despite elevated HR (∼120 beats·min^-1^). Thus, one possible explanation for the absence of cognitive improvement in the EMS + EX intervention is that brain activity associated with voluntary exercise was insufficient to induce cognitive improvement.

Decades of research has explored the physiological mechanisms underlying cognitive improvement during/after acute exercise. These include the separate and combined effects of neuromodulation by neurotransmitters and neurotrophic factors, cerebral blood flow, cerebral oxygenation, and cerebral metabolism (Basso and Suzuki, 2017; Ando et al., 2020; McMorris, 2021; Sudo et al., 2022; Williams et al., 2024). However, to date, the exact physiological mechanism(s) responsible for cognitive improvement remain unclear. We have recently provided novel evidence that suggests endogenous dopamine release plays a key role in cognitive improvement in response to acute exercise (Ando et al., 2024). Interestingly, this study has also shown that cognitive performance was only improved when exercise was associated with central signals from higher brain centers. The present findings provide further evidence to support the contribution of brain activity, associated with voluntary exercise, to cognitive improvement in that the amount of central neural activity is crucial to improve cognitive performance in response to acute exercise. Based on the inverted-U theory, which speculates on the relationship between exercise intensity and cognitive performance (Lambourne and Tomporowski, 2010; Chang et al., 2012; McMorris, 2021), the present findings indicate that increases in arousal did not reach optimal level that is required to lead to cognitive improvement when central neural activity was insufficient. Although this may, at least in part, explain the current findings, our data suggest further research is necessary to prove or refute this line of reasoning using sophisticated brain imaging techniques.

In our previous study, cognitive performance improved following the combination of EMS and arm cranking (Ando et al., 2024). One may argue that the present findings are contradictory to these previous findings. In both studies, HR was maintained at the same level (i.e., 120 beats·min^-1^) when EMS was combined with voluntary arm cranking (Ando et al., 2024) or leg cycling (present study). In our previous work, we demonstrated an improvement in RT following the combination of EMS and arm cranking (Ando et al., 2024). In both studies, HR was maintained at the same level (i.e., ∼120 beats·min^-1^) when EMS was combined with voluntary arm cranking (Ando et al., 2024) or upright cycling (present study). While it could be argued that these results are contradictory, the findings of Hill and others (2019) may offer additional mechanistic insight into these differences. They reported cognitive improvement following both arm-cranking and leg-cycling at 50% ergometer-specific maximal power output, but not following leg-cycling at the same absolute intensity as arm cranking (Hill et al., 2019). These combined results could suggest that brain activity is greater during arm cranking than leg cycling, when absolute exercise intensity was comparable. This theory may be corroborated by the fact that arm cranking exhibited a greater cerebral oxyhemoglobin level, compared to leg cycling, during exercise at the same relative intensity (Hashimoto et al., 2021). Furthermore, i) arm cranking is known to be less metabolically efficient compared with leg cycling (Kang et al., 1997), ii) cardiovascular strain is much larger despite a lower cardiac output (Calbet et al., 2015), iii) adrenaline concentration is higher after arm cranking at the same relative intensity (Leicht et al., 2017), and iv) more type II fibers may be recruited during arm cranking when compared to leg based cycling (Kang et al., 1997; Schneider et al., 2002). Collectively, this suggests that a combination of EMS and arm cranking is likely to be more ‘*effortful’* relative to a combination of EMS and leg cycling. Given that brain activity associated with central command appears to increase in an effort-dependent manner (Ishii et al., 2018), it can be speculated that greater central command was required to maintain the same HR when EMS is combined with arm cranking vs. leg cycling. These findings imply that exercise pressor reflex is likely to be different between EMS when combined with arm cranking and EMS when combined with leg cycling.

Alternatively, in our previous study, EMS was applied to inactive muscles during arm cranking (Ando et al., 2024). Conversely, in the present study, EMS was added to active muscles during cycling. EMS continuously activates lower limb muscle groups, including agonist and antagonist muscles. Hence, muscle contraction induced by EMS could support cycling to some extent, but it also interferes with muscle contractions during cycling. Although it remains unclear how the interference may affect central neural activity, we cannot rule out the possibility that cognitive performance was influenced by this interaction. In the present study, we did not evaluate the degree of muscle activities in the EMS + EX intervention and resultant influence on the brain activity. Furthermore, we determined exercise intensity based on HR, and mechanical efficiency was not evaluated. Therefore, further studies using neuroimaging (e.g., functional magnetic resonance imaging) and electromyographic techniques, together with metabolites and oxygen uptake measurements, would be warranted for better understanding of the present results.

EMS appears to randomly recruit motor unit (Bickel et al., 2011; Maffiuletti et al., 2011) or preferentially recruit type II fibers (Moritani, 2021). EMS is known to increase blood lactate (Hamada et al., 2003; Miyamoto et al., 2018; Kimura et al., 2019) and brain derived neurotrophic factor (BDNF) (Miyamoto et al., 2018; Kimura et al., 2019; Nishikawa et al., 2023) concentrations. It has been suggested that cognitive improvement following acute exercise may be linked with peripheral blood lactate (Tsukamoto et al., 2016; Hashimoto et al., 2018; Herold et al., 2022) or BDNF (Lee et al., 2014; Hwang et al., 2016) concentrations. Thus, one may argue that altered blood lactate and BDNF could play a role in the present results. However, we did not observe changes in cognitive performance in the EMS + EX intervention. Furthermore, a previous study indicated that EMS alone did not change cognitive performance despite increases in blood lactate and BDNF concentrations (Miyamoto et al., 2018). Thus, although we did not measure blood lactate and BDNF concentrations directly, it is less likely that changes in blood lactate and BDNF concentrations affected cognitive performance at least in the EMS + EX intervention.

Finally, this study is not without limitation. First, although we calculated sample size based on our previous data, we have to acknowledge that the number of participants was relatively small. Furthermore, we recruited only healthy young participants. Future research needs to consider larger sample sizes to draw firmer conclusions and make the present findings more applicable to the general population, including older individuals and/or patient groups. Second, we did not assess physical fitness (e.g., peak oxygen uptake) of the participants and it has been suggested that physical fitness level is one of the factors that influence acute exercise-cognition interaction (Lambourne and Tomporowski, 2010; Chang et al., 2012; McMorris, 2021; Sudo et al., 2022). Although the participants acted as their own controls in this randomized controlled crossover study, further studies are needed to clarify the combined effects of EMS and voluntary exercise on cognitive performance in relation to physical fitness level.

## 5 Conclusion

The present results indicate that a combination of EMS and cycling exercise does not alter cognitive performance despite elevated HR, equivalent to a moderate exercise intensity. Collectively, the present findings suggest that brain activity during EMS with cycling exercise may be insufficient to improve cognitive performance when compared to exercise alone.

## Data Availability

The raw data supporting the conclusion of this article will be made available by the authors, without undue reservation.
